# Supplementary figures and images for: A computational study of co-inhibitory immune complex assembly at the interface between T cells and antigen presenting cells
Source: PLoS Comput Biol. 2021 Mar 8;17(3):e1008825. doi: 10.1371/journal.pcbi.1008825 (PMC7971848; doi:10.1371/journal.pcbi.1008825)

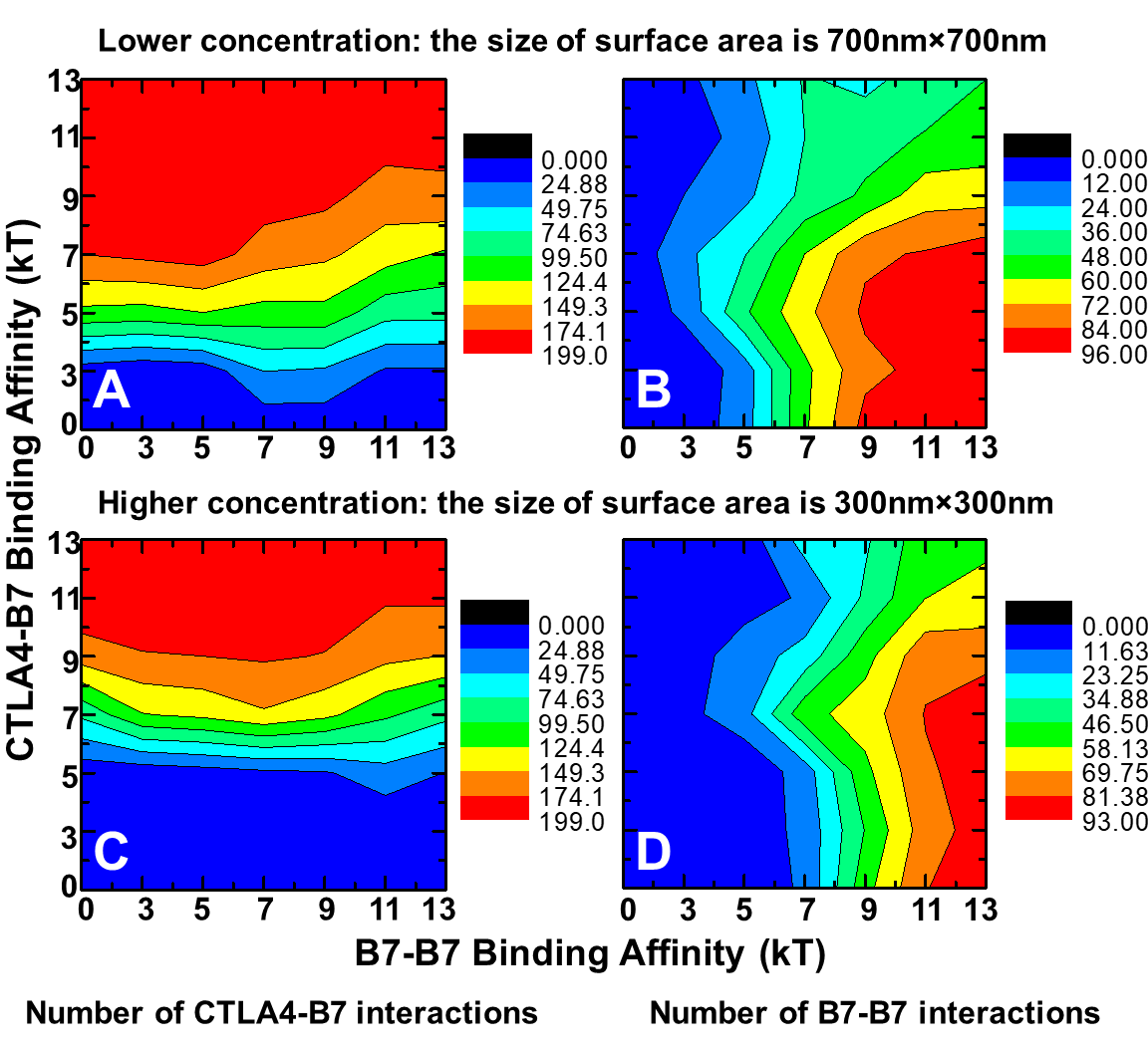

Supplement: S1 Fig — The contours in the two-dimensional heat maps indicate the number of CTLA-4/B7 trans-interactions (A) and the number of B7-B7 dimers (B) formed in the system with the lower concentration. The contours in the two-dimensional heat maps indicate the number of CTLA-4/B7 trans-interactions (C) and the number of B7-B7 dimers (D) formed in the system with the higher concentration. Detailed color indices are listed on the right-hand sides of each map. The x and y axes represent the values of two binding affinities. (TIF) [file pcbi.1008825.s001.tif]

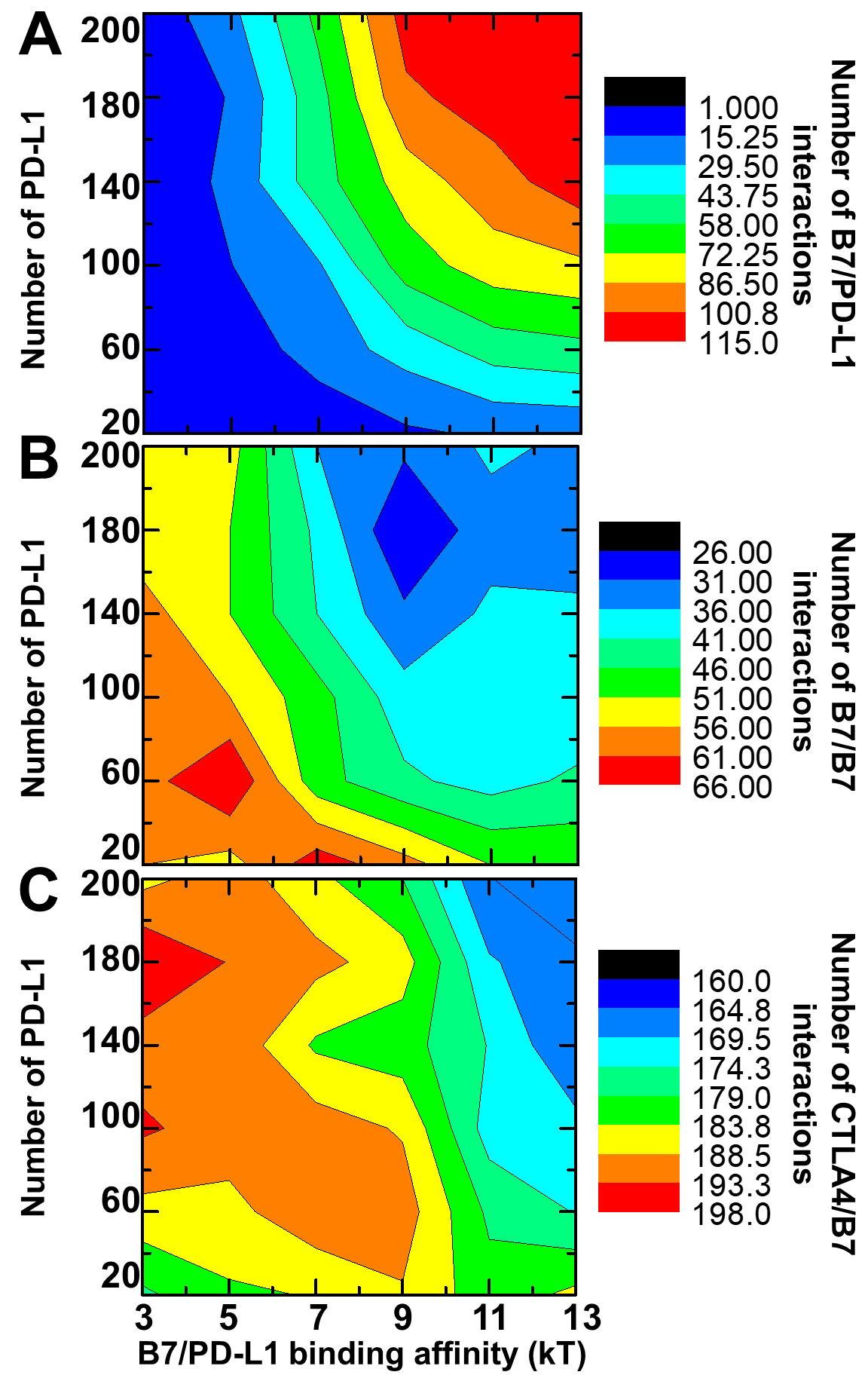

Supplement: S2 Fig — We systematically changed the surface density of PD-L1 and the binding affinity of its cis-interaction with B7. The tested results are summarized as two-dimensional heat maps. The contours in the maps indicate the number of PD-L1/B7 cis-interactions (A), the number of B7-B7 dimers (B) and the number of CTLA-4/B7 trans-interactions (C), respectively. Detailed color indices are listed on the right-hand sides of each map. The x axis represents the values of cis-binding affinity, and the y axis indicates the number of PD-L1 on the APC surface. (TIF) [file pcbi.1008825.s002.tif]

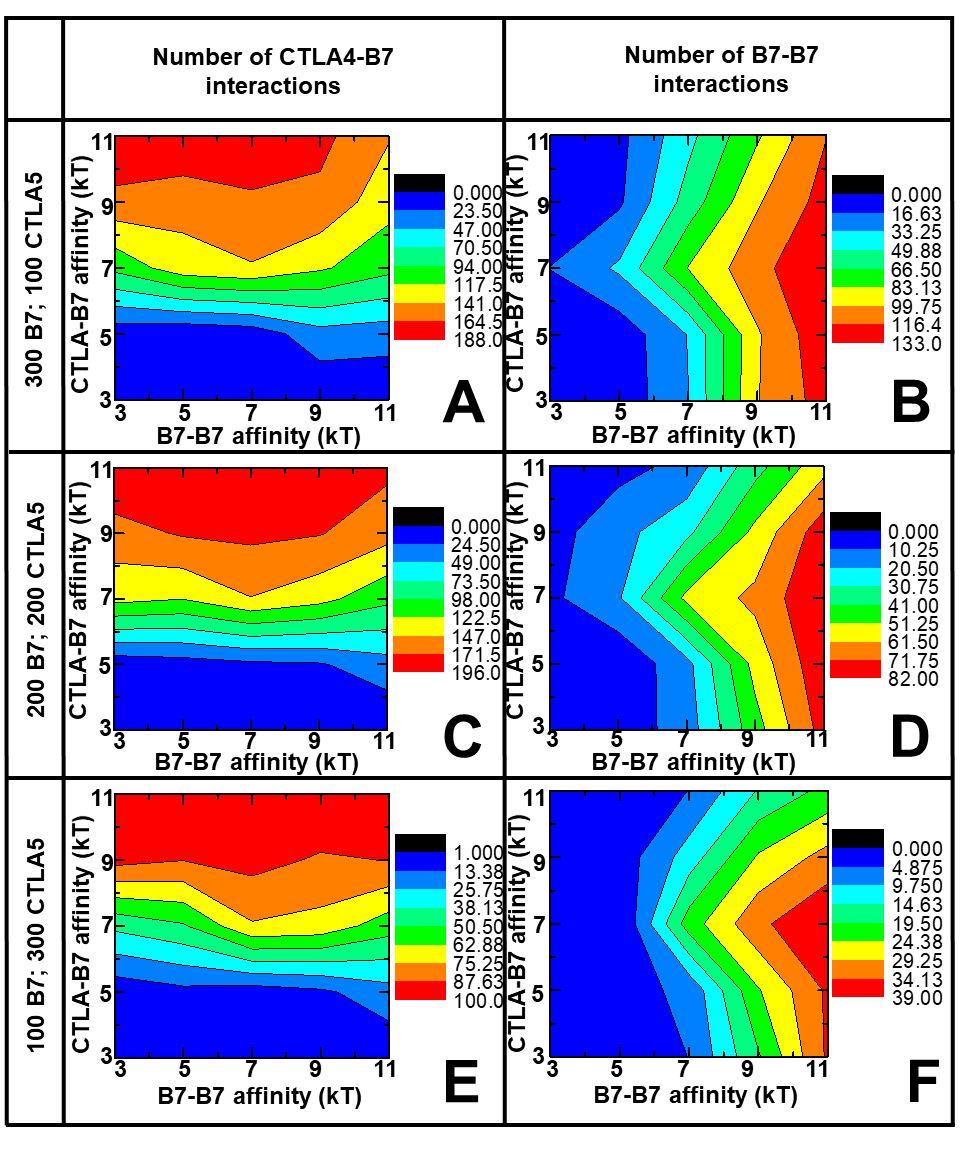

Supplement: S3 Fig — Under each ratio, the binding affinities of both CTLA-4/B7 trans-interaction and B7 homo-dimerization were changed from -3kT to -11kT with an interval of 2kT. The number of CTLA-4/B7 trans-interactions and the number of B7-B7 dimers formed in the system containing 300 B7 monomers and 100 CTLA-4 dimers are shown in (A) and (B) as 2D colorful heat map. Different combinations of binding affinities are indexed by the y and x aces of the maps. Similarly, the number of CTLA-4/B7 trans-interactions and the number of B7-B7 dimers formed in the system containing 200 B7 monomers and 200 CTLA-4 dimers are shown by (C) and (D). Finally, the number of CTLA-4/B7 trans-interactions and the number of B7-B7 dimers formed in the system containing 100 B7 monomers and 300 CTLA-4 dimers are shown by (E) and (F). Detailed color indices are listed on the right-hand sides of each map. (TIF) [file pcbi.1008825.s003.tif]
