# Supplementary material for: A computational study of co-inhibitory immune complex assembly at the interface between T cells and antigen presenting cells
Source: PLoS Comput Biol. 2021 Mar 8;17(3):e1008825. doi: 10.1371/journal.pcbi.1008825 (PMC7971848; doi:10.1371/journal.pcbi.1008825)
Supplement: Attachment — Submitted filename: PD1B7cis_Response_to_Reviewers.docx [file pcbi.1008825.s004.docx]

**Responses to Reviewer's Questions**

******************************************************************************

Reviewer #1:

In the paper "A Computational Study of Co-inhibitory Immune Complex Assembly at

the Interface between T cells and Antigen Presenting Cells", Su et al develop a coarse-grained mesoscale model of protein-ligand and protein-protein interactions in order to elucidate their mechanisms of assembly in T cells.

The main novelty of the approach relies in accounting for different binding interfaces and kinetic rates, which give rise to different steady state properties of the system. The author carry on a systematic parameter scan in order to evaluate the relative importance on these parameters on the amount and relative types of interactions.

While the paper is interesting and the result are novel I recommend three minor modifications:

1) a more systematic and quantitative comparison of the obtained results with experiments. This comparison is often implied, but a quantitative description would strengthen the conclusions.

- We thank our reviewer for this constructive suggestion. We have now provided more detailed description to connect our simulation results with previous experiments throughout the paper. For instance, on page 16 of revised manuscript, we have added that “it is worth mentioning that this negative coupling between CTLA-4/B7 engagement and B7/PD-L1 cis-interaction has also been observed by recent experiments. In the experiment, the split nano-luciferase constructs were generated by cloning full-length B7 and PD-L1 into LgBiT or SmBiT vectors. The HEK 293 freestyle cells were co-transfected with these constructs. As a result, an approximately two-fold reduction of the B7 SmBit: PD-L1 LgBit luminescence was obtained in the presence of either soluble CTLA-4 hIgG1 protein or CTLA-4 GFP-expressing cells, confirming our simulation results that CTLA-4/B7 engagement and B7/PD-L1 cis-interaction is negatively coupled”. Moreover, on page 20 of revised manuscript, we have added that “our statistical results thus suggest that the signaling of both co-inhibitory receptors CTLA-4 and PD-1 can be negatively regulated by the lateral crosstalk between their ligands (B7 and PD-L1). This conclusion is supported by previous experimental evidences [15, 50]. In the experiments, Raji B cells were first stained by CTLA-4-Fc in a dose dependent manner. It was found that co-expression of PD-L1 with 5.9-fold excess to B7 significantly reduced CTLA-4-Fc staining, leading into an approximately 4.7-fold higher dissociation constant. This result suggests that the original B7/CTLA-4 interaction can be inhibited by the cis-interaction between B7 and PD-L1. Vice versa, when PD-L1 transduced, CD80 knockout Raji B cells were stained by PD-1-Fc in a concentration dependent manner, this PD-1-Fc staining was dramatically decreased by co-expression of CD80 with 3.5-fold excess of PD-L1. This result suggests that the PD-1/PD-L1 trans-interaction can also be inhibited by the cis-interaction between B7 and PD-L1”. Finally, we have added more detailed experimental evidence to support our model assumption than a dimer of CTLA-4 can bind to two B7 monomers simultaneously. As shown on page x of revised manuscript, we mentioned that “based on previous experimental evidences in the crystal structure of the human CTLA-4/B7-1 co-stimulatory complex (PDB id 1I8L), CTLA-4 exists as homodimers on T cells through an interface of highly conserved residues. Each CTLA-4 in the dimer further binds to a B7 monomer simultaneously, providing the structural basis of a zipper-like oligomerization”.

2) A careful editing of the paper. A number of sentences would benefit from a grammar check.

- We thank our reviewer for this suggestion. We have now carefully edited the manuscript and corrected typo and grammatical errors as best as we could. For instance, “In another word” on both page 17 and page 20 of the original manuscript have been changed into “In other words”. “even the number of PD-L1 was increased from 100 to 180” in page 15 of the original manuscript has been changed into “even though the number of PD-L1 was increased from 100 to 180”. “incorporate these factors into our simulations will add another layer of complexity to the spatial organization of coregulatory signaling” in page 6 of the original manuscript has been changed into “the incorporation of these factors into our simulations will add another layer of complexity to the spatial organization of coregulatory signaling”. In the original caption of Figure 4, "In addition to CTLA-4 and B7, ligand PD-L1, we further introduced PD-L1 into the simulation system." has been changed into "In addition to CTLA-4 and B7, we further introduced PD-L1 into the simulation system".

3) A few general sentences in the introduction about the mechanisms of T cells assembly at interface would better familiarize the reader with the specific topic of the paper.

- We thank our reviewer for making this comment. We have now added more background about the receptor assembly at interface between T cell and APC in the introduction section. As shown on page 5 of revised manuscript, “it is likely that the combination of their trans and cis-interactions further drive the aggregation of these ligand-receptor complexes into higher-order oligomers at the interface between T cell and APC. As observed in previous experiments, co-regulatory receptors such as CTLA-4 and CD28 are spatially co-localized together with TCR/MHC complexes after the initial encounter of T cells with APC, leading into the assembly of signaling hubs called micro-clusters. After further spreading of the T cell/APC interface, these micro-clusters merge together into a ring-like structure called central supramolecular activation cluster (cSMAC), as part of the immunological synapse. Like many other systems of ligand-receptor complexes, formation of these spatial-temporal patterns at the interface between T cells and APC plays an essential role as the intercellular platform to regulate the dynamics of immune signaling pathways”.

******************************************************************************

Reviewer #2:

The manuscript by Su et al. reports a coarse-grained reaction-diffusion simulation model of T cell receptors, CTLA-4 and PD-1, and their corresponding antigen presenting cell (APC) ligands, B7 and PD-L1. By varying the binding constants in the model, the authors showed that cis-interactions between the two ligands on the APC membrane can inhibit CTlA-4/B7 oligomerization because of the competition for an overlapped B7 binding and homodimerization site. The introduction of PD-1 into the system reduces the inhibition by competing for the PD-L1 binding site that is shared with B7. The authors suggest these competitive bindings can regulate the signalling pathways activated by B7 and PD-L1.

To be acceptable for publication, the authors should address the following issues:

1. The simulation model assumes that a dimer of CTLA-4 can bind to two B7 monomers simultaneously. Reference to previously reported structural or other experimental evidence that support this assumption should be provided.

- We thank our reviewer for the suggestion. We apologize for not providing the reference. We have now cited the original paper about the CTLA-4/B7 complex: “Stamper CC, Zhang Y, Tobin JF, Erbe DV, Ikemizu S, Davis SJ, Stahl ML, Seehra J, Somers WS, Mosyak L. Crystal structure of the B7-1/CTLA-4 complex that inhibits human immune responses. Nature. 2001 Mar 29; 410(6828): 608-11”. Moreover, we have added more detailed background description about this reference in the main text as experimental evidence to support our model. Specifically, we mentioned that “based on previous experimental evidences in the crystal structure of the human CTLA-4/B7-1 co-stimulatory complex (PDB id 1I8L), CTLA-4 exists as homodimers on T cells through an interface of highly conserved residues. Each CTLA-4 in the dimer further binds to a B7 monomer simultaneously, providing the structural basis of a zipper-like oligomerization. Because the homo-dimerization of CTLA-4 does not interfere with any other interactions in this study, we assume that CTLA-4 dimers preform in the system to avoid further complication”. Please see page 7 of revised manuscript for details.

2. The translational diffusion coefficient of the ligand-receptor complex was set to 5 um2/s but for ligand-ligand hetero- and homo-dimers, the coefficient is 0 um2/s. Why the discrepancy? Would letting all dimers, whether they are ligand-receptor or ligand-ligand, diffuse at 5 um2/s change any of the results presented in the manuscript?

- We thank our reviewer for asking this question. We have now provided more detailed discussions about why the diffusion coefficient of cis-dimers was set to 0 in our model. As shown in page 9 of revised manuscript, we wrote that “after the formation of a trans-interaction, the ligand-receptor complex diffuses together at the cellular interface with a relatively lower diffusion coefficient. In contrast, we assume that if the interaction is formed between two proteins on the same cell surface, they will stop diffusing. This assumption was made to facilitate lateral oligomerization of ligand-receptor complexes. It is based on the following consideration. Previous single molecule imaging experiment showed that diffusions of membrane proteins on the same cell surface can be reduced by at least an order of magnitude upon oligomerization [ref]. Moreover, diffusions were even slower as the size of oligomers became larger. It was proposed that these oligomers of ligands or receptors formed through the cis-interactions are trapped due to enhanced connections between their intracellular regions and cytoskeleton. Therefore, although the mobility of entire oligomers was still detectable in the experiment, they are set as static in our simulation to avoid computational complexity. We believe this simplification will not significantly affect our simulation outputs”.

3. In the model, the ratio of CTLA-4 to B7 is always fixed to 1:1, which is likely not the case in vivo. The authors should show how does the correlation between CTLA-4/B7 trans-interaction and B7 homodimerization in Figure 3 change when the ratio is changed.

- We thank our reviewer for this constructive suggestion. We have now carried out additional tests under different ratios of CTLA-4 to B7. Specifically, three systems were compared. The first system contains 300 B7 monomers and CTLA-4 dimers; the second system contains 200 B7 monomers and 200 CTLA-4 dimers; while the third system contains 100 B7 monomers and 300 CTLA-4 dimers. In each system, the binding affinities of both CTLA-4/B7 trans-interaction and B7 homo-dimerization were changed from -3kT to -11kT with an interval of 2kT. A total number of 5×5=25 combinations were tested. The overall results are summarized as two-dimensional heat maps in Figure S3. The number of CTLA-4/B7 trans-interactions and the number of B7-B7 dimers formed in the first system are shown by Figure S3a and Figure S3b, respectively. The number of CTLA-4/B7 trans-interactions and the number of B7-B7 dimers formed in the second system are shown by Figure S3c and Figure S3d, respectively. The number of CTLA-4/B7 trans-interactions and the number of B7-B7 dimers formed in the third system are shown by Figure S3e and Figure S3f, respectively. Detailed color indices are listed on the right-hand sides of each map. Figure S3 indicates that the patterns in all these systems are very similar. The subtle difference is manifested in the fact that is specified as follows. The number of CTLA-4/B7 interactions under strong binding affinities (upper-right corners of the maps in Figure S3a, S3c and S3e) shows the most negative correlation in the first system and the weakest negative correlation in the third system. In contrast, the number of B7 homo-dimers under strong binding affinities (upper-right corners of the maps in Figure S3b, S3d and S3f) shows the most negative correlation in the third system and the weakest negative correlation in the first system. This can be explained as follows. If the number of B7 monomers is higher than the number of CTLA-4 dimers, the dimerization of B7 monomers will dominate the system and thus cause stronger impacts on CTLA-4/B7 interactions as reflected by the map in Figure S3a. On the other hand, if the number of B7 monomers is lower than the number of CTLA-4 dimers, the CTLA-4/B7 interactions will dominate the system and thus cause stronger impacts on dimerization of B7 monomers as reflected by the map in Figure S3f. Above discussions have been added at page 13-14 of the revised manuscript. Moreover, Figure S3 is included in the newly prepared Supporting Information.

There were some language issues, for example:

page 6: "Incorporate these factors into.."

- We thank our reviewer for pointing out this typo. The grammatical error in the original manuscript has now been changed as follows: “the incorporation of these factor into our simulations will add another layer of complexity to the spatial organization of coregulatory signaling”. Please see page 7 of revised manuscript for details.

page 15: "-3kT, even the number..."

- We thank our reviewer for pointing out this typo. “even the number of PD-L1 was increased from 100 to 180” in the original manuscript has been changed into “even though the number of PD-L1 was increased from 100 to 180”.

page 17: "In another word,..."

- We thank our reviewer for pointing out this typo. Both “In another word” on page 17 and page 20 in the original manuscript have been changed into “In other words”.

page 28: Caption of Figure 4: "In addition to CTLA-4 and B7, ligand PD-L1, we further introduced PD-L1 into the simulation system."

- We thank our reviewer for pointing out this mistake. We have now removed the redundancy in the original figure caption. The revised caption of Figure 4 is as follows: “in addition to CTLA-4 and B7, we further introduced PD-L1 into the simulation system”.
